# The Utility of Radiologic Imaging and Full Body Skin Examination in Patients With Melanoma of Unknown Primary

**DOI:** 10.7759/cureus.58523

**Published:** 2024-04-18

**Authors:** Parisa ShamaeiZadeh, Elliott Campbell, Nneka Comfere

**Affiliations:** 1 Dermatology, University of Kentucky College of Medicine, Lexington, USA; 2 Dermatology, Mayo Clinic, Rochester, USA; 3 Laboratory Medicine and Pathology, Mayo Clinic, Rochester, USA

**Keywords:** melanoma, dermatology, tumoral melanosis, pet-ct, melanoma of unknown primary

## Abstract

Although most melanomas have a cutaneous origin, melanomas are rarely discovered without an overt primary site and are found in the metastatic stage. This phenomenon is called melanoma of unknown primary (MUP), which was first recorded in 1963.Melanoma can also rarely present as tumoral melanosis, which has completely regressed. By definition, this does not have viable melanocytes and histologically presents as an infiltration of melanophages and melanin. A 71-year-old female presented for dermatologic evaluation after being found to have melanoma of unknown primary (MUP). The MUP, located in multiple lymph nodes of the left superior and inferior inguinal region, was found on preoperative imaging indicated for surgical management of endometrial carcinoma. After the biopsy, a positron emission tomography-computed tomography (PET-CT) scan was performed to determine the extent of involvement, which noted focal uptake of the left heel of just medial to midline with an SUV max of 2.1. Based on the PET-CT findings, the patient was questioned about the lesion on her heel. She had suspected this was due to friction and stated it had been asymptomatic and present for years.

This unique case demonstrates that combined total skin examination and whole-body radiologic imaging (preferably PET-CT) are both critical elements in the evaluation of MUP. Since melanoma of unknown primary is at least American Joint Committee on Cancer (AJCC) 8 Stage III (due to N1 status), imaging is reasonable in these patients.

## Introduction

Malignant melanoma is a neoplasm that has morbidity and mortality associated if not managed early, underscoring the need to identify early signs of disease [1].

Although most melanomas have a cutaneous origin, melanomas are rarely discovered without an overt primary site and are found in the metastatic stage. This phenomenon is called melanoma of unknown primary (MUP), which was first recorded in 1963 [2]. The hypotheses surrounding the phenomenon of MUP include spontaneous regression of the primary site, the presence of ectopic melanocytes, or primary sites in unusual anatomic locations (e.g. gastrointestinal tract) [3]. In instances where a patient is deemed to have “melanoma of unknown primary,” it is critical for the dermatologist to perform a detailed review of systems and skin exams to determine signs of widespread involvement and attempt to find a primary site. Melanoma can also rarely present as tumoral melanosis, which is a rare, completely regressed melanoma. By definition, this does not have viable melanocytes and histologically presents as an infiltration of melanophages and melanin [4]. Here, we report a rare case of tumoral melanosis alongside findings of metastatic melanoma in the setting of MUP discovered following a positron emission tomography-computed tomography (PET-CT) scan for endometrial carcinoma. This case emphasizes the need to consider full body imaging in conjunction with clinical skin examinations for the evaluation of MUP.

## Case presentation

**Figure 1:**
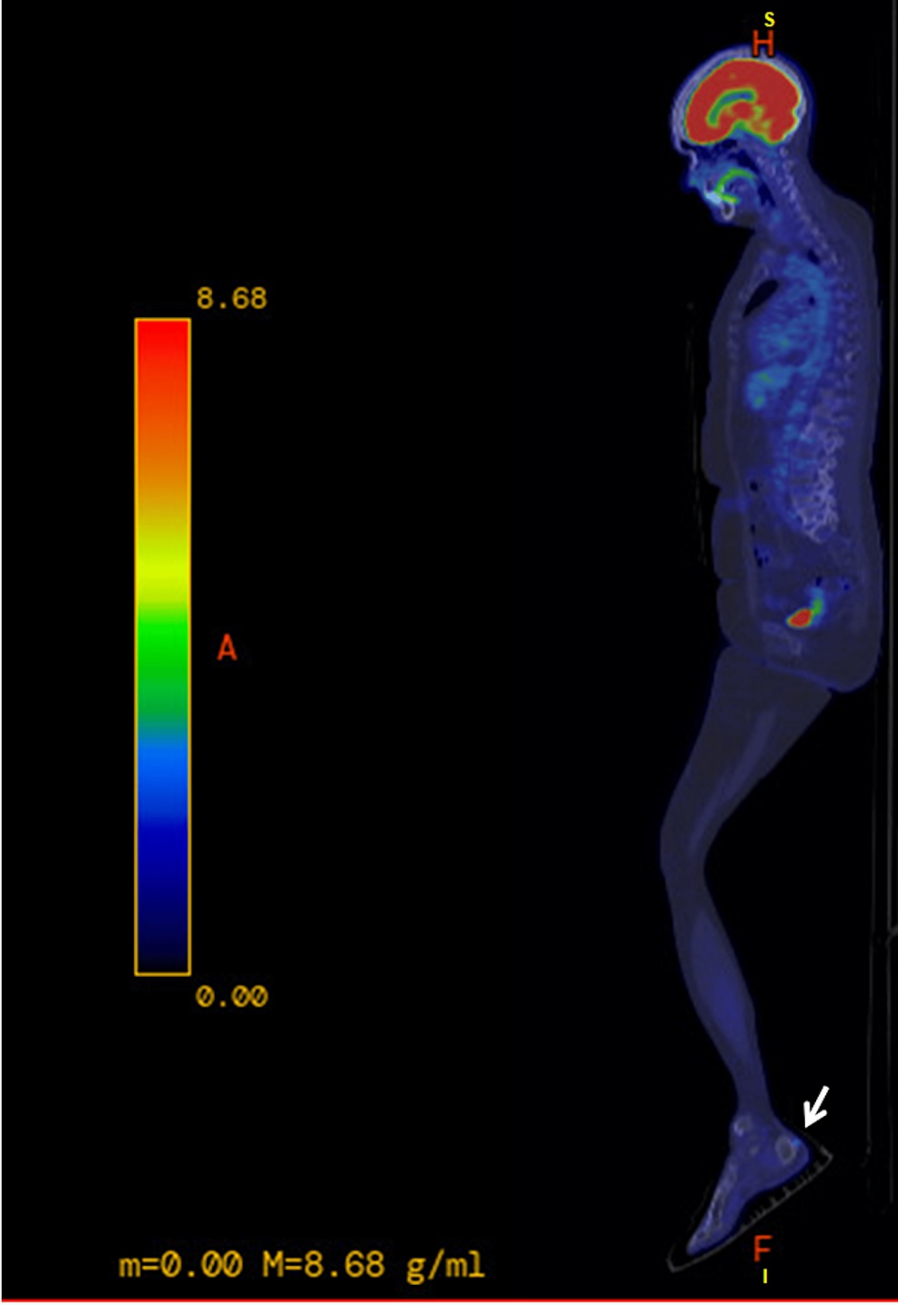
PET-CT scan results noting focal uptake of the left heel just medial to midline PET-CT: positron emission tomography-computed tomography

A 71-year-old female presented for dermatologic evaluation after being found to have MUP. The MUP, located in multiple lymph nodes of the left superior and inferior inguinal region, was found on preoperative imaging indicated for surgical management of endometrial carcinoma on March 7, 2023. After the biopsy, a PET-CT scan was performed to determine the extent of involvement, which noted focal uptake of the left heel of just medial to midline with an SUV max of 2.1 (Figure 1). Based on the PET-CT findings, the patient was questioned about the lesion on her heel. She had suspected this was due to friction and stated it had been asymptomatic and present for years.

**Figure 2:**
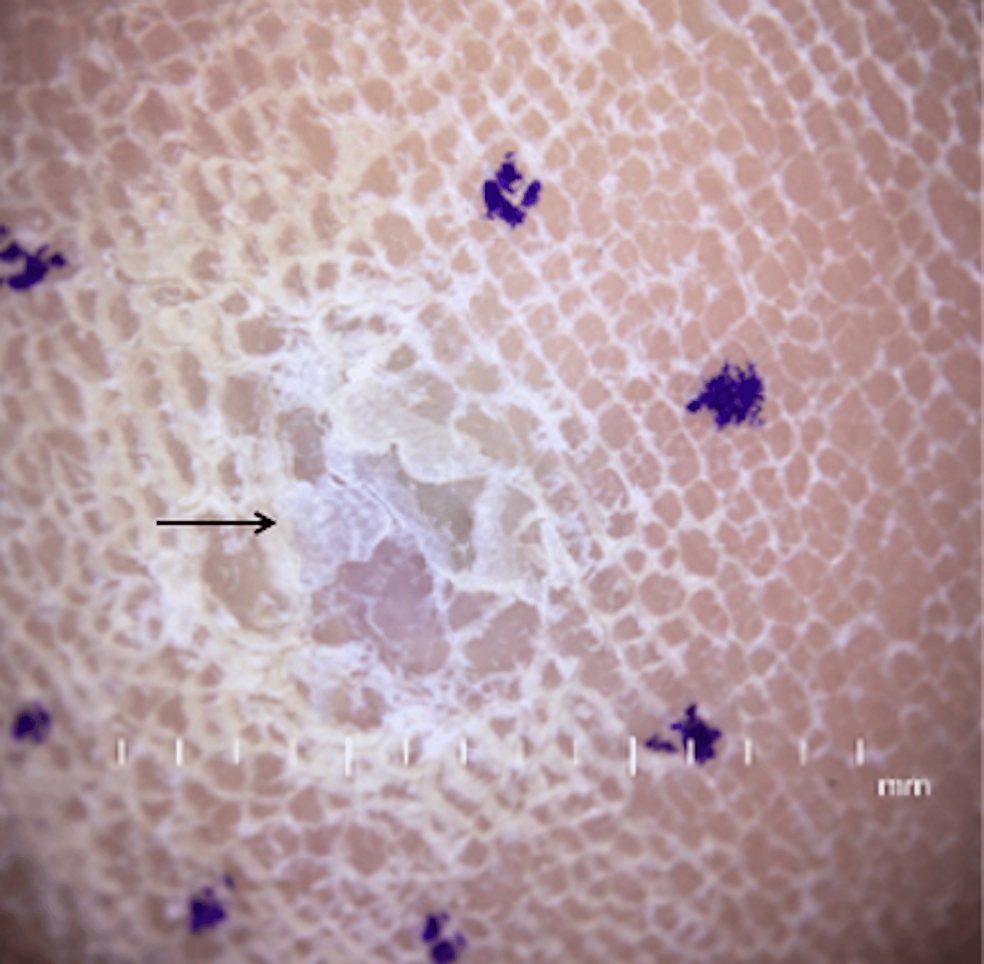
The left plantar heel with dermoscopic imaging A slightly deep, darkened hue can be appreciated under the keratin.

**Figure 3:**
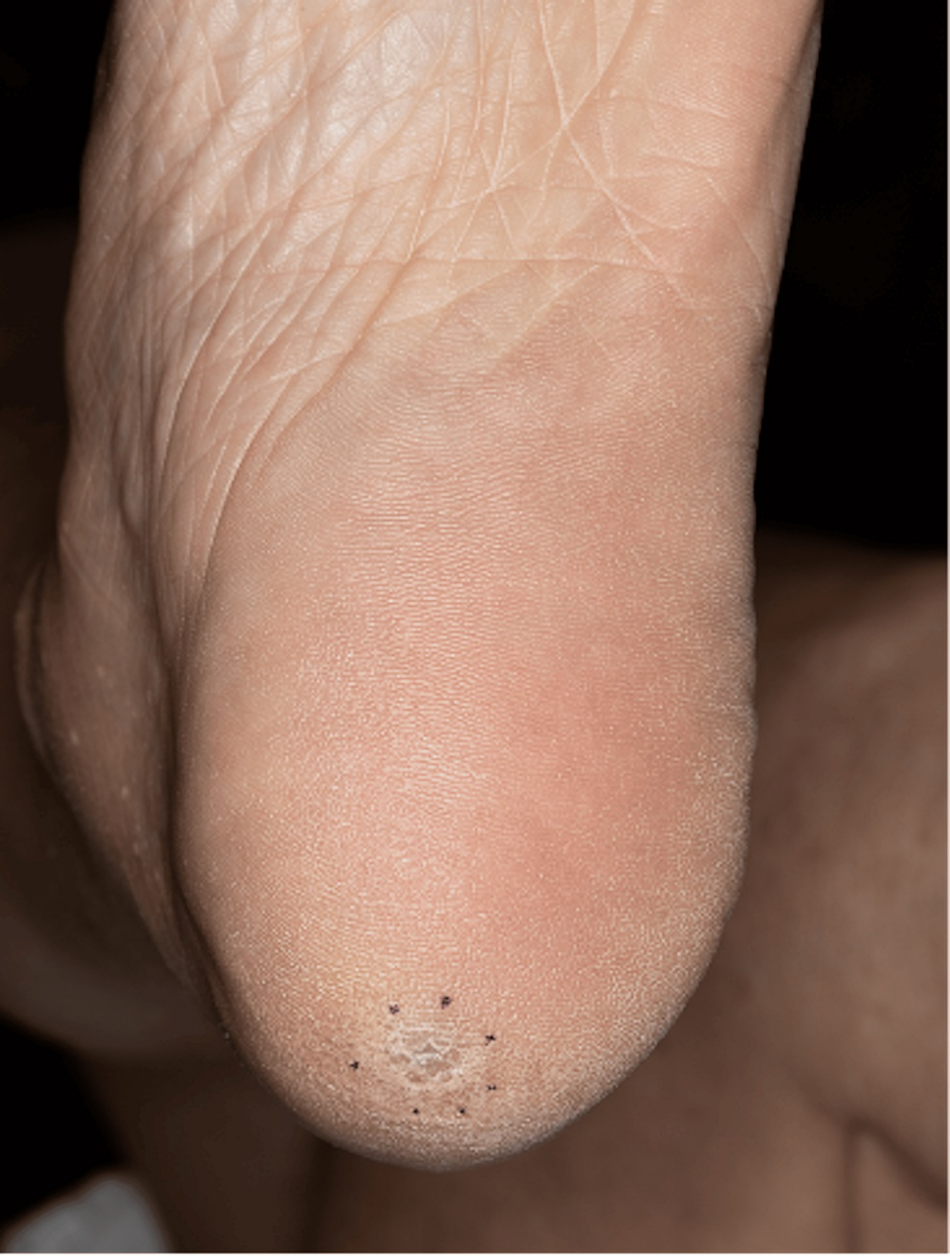
The left plantar heel Hyperkeratotic plaque with a subtle deep blue hue underlying the keratin.

**Figure 4:**
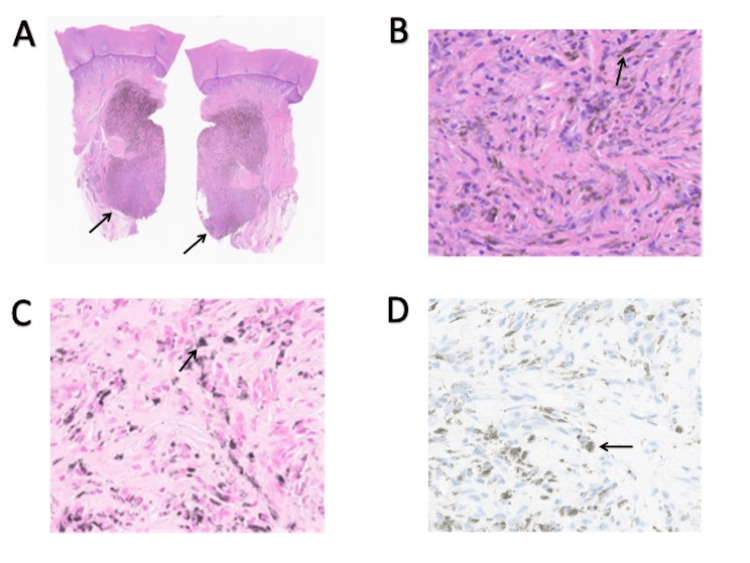
Panel A: Skin biopsy demonstrating a nodular dermal-based mass with pigmentation on H&E, 1.25x magnification; Panel B: Demonstration of melanophages without viable melanocytes on H&E, 20x magnification; Panel C: Fontana Masson demonstrating pigmentation on 20x magnification; Panel D: Melan A highlights a lack of viable melanocytes on 20x magnification

Physical examination revealed an 8 mm plaque on the left plantar heel, clinically appearing consistent with a callus (Figures 2-3). A biopsy was performed based on the PET-CT findings and a small amount of potential darkening under the keratin layer centrally. A punch biopsy of the left plantar heel revealed extensive melanin pigment within melanophages without viable melanocytes, consistent with tumoral melanosis (Figure 4). This was suspected to be primary melanoma, given the location and timeline. After a multidisciplinary discussion with dermatologic surgery, surgical oncology, and oncology, the decision was made to not excise the lesion since there was no viable melanoma on the initial biopsy. The patient was ultimately initiated on immunotherapy with nivolumab. After completing one year of immunotherapy, a repeat PET-CT found no evidence of fluorodeoxyglucose (FDG)-avid recurrent or metastatic disease.

## Discussion

This unique case demonstrates that combined total skin examination and whole-body radiologic imaging (preferably PET-CT) is a critical element in the evaluation of MUP. Since melanoma of unknown primary is at least American Joint Committee on Cancer (AJCC) 8 Stage III (due to N1 status), imaging is reasonable in these patients. Although imaging is warranted in most cases of Stage III or higher melanomas for staging purposes, this work emphasizes the importance of obtaining this prior to dermatologic evaluation, when feasible. If PET-CT demonstrates uptake in the skin, there should be a high index of suspicion for melanoma, regardless of clinical appearance. Given the paucity of guidelines and management literature regarding MUP, patients should be treated based on current guidelines for a similar stage, known as primary melanoma (which, by definition, will be Stage III or higher) [3]. Although there is no measured incidence in the literature, in the author's experience at a tertiary referral institution, true melanoma of unknown primary is seen extremely infrequently (less than monthly). Some authors theorize that some instances of MUP are partially regressed primary melanoma; however, this has not been substantiated [5]. This case also demonstrates that tumoral melanosis may be the presenting and residual sign of a preceding melanoma that has fully regressed. The understanding of the prognosis of tumoral melanosis remains limited. Existing literature indicates that focal regression within melanoma does not significantly impact morbidity or mortality. Nevertheless, extensive regression in larger lesions may be linked to an unfavorable prognosis [6-9]. Therefore, the discovery of tumoral melanosis in a patient without a known history of melanoma should trigger a comprehensive assessment for metastatic disease [10]. There is no evidence that the authors are aware of that defines the sensitivity of imaging for tumoral melanosis. The regression of this melanoma likely made it inconspicuous, which ultimately led to a metastatic lesion in the lymph nodes.

## Conclusions

This case emphasizes the significance of a comprehensive skin examination complemented by radiologic imaging and a review of systems in the evaluation of patients with melanoma of unknown primary. The case's resemblance to a callus highlights the potential challenge in diagnosing regressed melanomas (in this case, tumoral melanosis) on standard skin examination alone.
